# Ibutilide protects against cardiomyocytes injury via inhibiting endoplasmic reticulum and mitochondrial stress pathways

**DOI:** 10.1007/s00380-016-0891-1

**Published:** 2016-09-17

**Authors:** Yu Wang, Yi-Li Wang, Xia Huang, Yang Yang, Ya-Jun Zhao, Cheng-Xi Wei, Ming Zhao

**Affiliations:** 1 Medicinal Chemistry and Pharmacology Institute, Inner Mongolia University for the Nationalities, No. 22 Holin He Street, Tongliao, Inner Mongolia 028002 People’s Republic of China; 2 Affiliated Hospital of Inner Mongolia University for Nationalities, No. 1472 Holin He Street, Tongliao, Inner Mongolia 028002 People’s Republic of China

**Keywords:** Ibutilide, Atial fibrillation, Oxidative stress, Endoplasmic reticulum stress, Mitochondrial stress

## Abstract

Atrial fibrillation (AF) is a complex disease with multiple inter-relating causes culminating in rapid atrial activation and atrial structural remodeling. The contribution of endoplasmic reticulum and mitochondria stress to AF has been highlighted. As the class III antiarrhythmic agent, ibutilide are widely used to AF. This study was designed to explore whether ibutilide could treat AF by inhibiting endoplasmic reticulum stress pathways and mitochondria stress. The neonatal rat cardiomyocytes were isolated and exposed to H_2_O_2_, ibutilide was add to the culture medium 12 h. Then the cell viability, oxidative stress levels and apoptotic rate were analyzed. In addition, endoplasmic reticulum stress related protein (GRP78, GRP94, CHOP), mitochondria-dependent protein (Bax, Bcl-2) and caspase-3/9/12 were identified by real-time PCR and western blot analysis. In our results, remarkable decreased cell viability and oxidative stress levels were detected in cardiomyocytes after treating with H_2_O_2_. The apoptotic rate and the expression of proteins involved in mitochondrial stress and endoplasmic reticulum stress pathways increased. While ibutilide significantly inhibited these changes. These data suggested that ibutilide serves a protective role against H_2_O_2_-induced apoptosis of neonatal rat cardiomyocytes, and the mechanism is related to suppression of mitochondrial stress and endoplasmic reticulum stress.

## Introduction

Atrial fibrillation (AF) is the most serious cardiovascular disease in the world [1]. Because of aging of the population and improved survival of patients with other cardiovascular diseases, the prevalence of AF is increasing during the past few decades. In United States, the population of AF is increasing, from 2.3 million patients currently to 5.6 million by the year 2050 [2]. AF and its complication, such as palpitation, drop of cardiac output, heart failure and so on, threaten people health and increase the risk of stroke in patients [3]. The pathophysiology of AF is complex. Accumulating evidence has implicated a potential role of reactive oxygen species (ROS) production which leads to oxidative stress and apoptosis in pathogenesis of AF [4]. During the injury, mitochondria and endoplasmic reticulum (ER) play important roles [5]. Production of ROS has been linked to ER stress and the unfolded protein response (UPR), ROS plays a critical role in many cellular processes and can be produced in the cytosol and several organelles, including the ER and mitochondria [6]. However, their relationship in neonatal rat cardiomyocytes is not clear.

Many treatments have been used in AF. Catheter radiofrequency ablation has been the most widely used and the most effective method to modify the substrate of AF. But this method is suitable for use in certain patient groups [7]. Chemical drugs are less invasive, more cost-effective, and, unlike catheter radiofrequency ablation, it does not require sedation [8]. Common drugs used for treatment AF contain ibutilide, propafenone, dofetilide and so on [9]. Ibutilide is class III antiarrhythmic drug. It was approved on December 28, 1995 by the Food and Drug Administration. Due to ibutilide extensive first-pass metabolism, it is available only for intravenous. Ibutilide can prolong repolarization time, action potential duration, and refractory period of ventricular and atrial myocardium through its action as a potassium channel blocker, affecting the rapid component of the cardiac delayed rectifier potassium current [10]. However, the molecular mechanisms of ibutilide protective effects are still not fully clarified.

To determine the mechanism of ibutilide on protect cardiomyocytes, the neonatal rat cardiomyocytes were isolated and cultured. The model in vitro of cardiomyocytes injury by neonatal rat cardiomyocytes under H_2_O_2_ treatment was created. Then the existence of mitochondrial and ER stress pathways in H_2_O_2_-induced neonatal rat cardiomyocytes apoptosis and the protection of ibutilide were confirmed.

## Materials and methods

### Materials

Super M-MLV reverse transcriptase was purchased from BioTeke. RNA simple total RNA Kit was acquired from TIANGEN (Beijing, China). Ibutilide and H_2_O_2_ were obtained from Sigma (American). Terminal deoxynucleotidyl transferase-mediated dUTP nick end-labeling (TUNEL) assay kit was purchased from Beyotime Biotechnology (ShangHai, China).

### Cell culture

Primary cultures of neonatal rat cardiomyocytes were obtained as described previously [11]. All experiments in this research were performed in accordance with the Guidelines of Animal Experiments from the Committee of Medical Ethics, National Health Department of China (1998). The cardiomyocytes were maintained in DMEM supplemented with 10 % FBS (fetal bovine serum) and 1 % penicillin and streptomycin under an atmosphere of 95 % air and 5 % CO_2_ at 37 °C. The medium was replaced every 3 days and cells were subcultured or subjected to experimental procedures at an amount to achieve 70–80 % confluency. H_2_O_2_ induced cytotoxicity at this concentration [12]. Ibutilide was present in the treatment group.

### Cell viability assay

Cytotoxicity of neonatal rat cardiomyocytes was tested by the a colorimetric assay using 3-(4,5)-dimethylthiahiazo (-z-yl)-3, 5-di-phenytetrazoliumromide (MTT) (Sigma-Aldrich). Cells were seeded into 96-well plated and exposed to DMEM or H_2_O_2_ within/without ibutilide. Experiments were done during the exponential phase of cell growth.

### Measurement of glutathione peroxidase (GSH-px), MDA and SOD activity in cardiomyocytes

The cellular activities of GSH-px, MDA and SOD were measured commercial assay kits according to the manufacturers’ instructions. Then they were determined by commercially available enzyme-linked immunosorbent assays (ELISAs). All samples were assayed at three times.

### TUNEL staining

DNA fragmentation of apoptotic cells was detected by TUNEL staining following the manufacturer’s instructions. After the cardiomyocytes were cultured for 24 h, the cells were fixed by 4 % paraformaldehyde solution for 30 min. Then 0.3 % H_2_O_2_ was used for block endogenous peroxidase activity in cells. Last TUNEL reaction was used to incubate cells. The results were detected by microscopy.

### Real-time PCR

The RNA of cardiomyocytes was isolated by RNA extraction kit (TIANGEN, Beijing, China), and then the RNA was transcribed. The obtained cDNA was used for real-time PCR.

The primers of GRP78, GRP94, GAPDH gene fragments were designed following:

- GRP78-F: 5′ GATAATCAGCCCACCGTAA 3′
- GRP78-R: 5′ TTGTTTCCTGTCCCTTTGT 3′
- GRP94-F: 5′ GATGTGGATGGCACGGTAG3′
- GRP94-R: 5′ GTTCCCTTATTTGTGATGCA 3′
- GAPDH F: 5′ CGGCAAGTTCAACGGCACAG 3′
- GAPDH R: 5′ CGCCAGTAGACTCCACGACAT 3′

Amplification was performed in duplicate on FTC-3000 Real-Time PCR system thermocycler using SYBR Green PCR Master Mix (TIANGEN, Beijing, China). The reaction condition was s 95 °C for 15 min and following 40 cycles: denaturation (95 °C for 10 s), annealing and elongation (60 °C for 60 s). The relative mRNA expression level of the gene was normalized to the level of GAPDH in the same sample.

### Western blot analysis

Western blot was performed as described previously [13]. The primary antibodies contain anti-caspase-3 (1:500), anti-caspase-9 (1:500), anti-caspase-12 (1:500) (Bioss), anti-CHOP (1:1000), anti-Bcl-2 (1:1000) (Wanleibio), and anti-GRP78 (1:400), anti-GRP94 (1:400), anti-Bax (1:400) (Boster).

### Statistical analysis

All values were expressed as mean ± SEM. Multi-group comparisons of the means were carried out by matched *t* test using SPSS 11.5.

## Results

### Ibutilide improved the cell viability of H_2_O_2_-induced neonatal rat cardiomyocytes

**Fig. 1:**
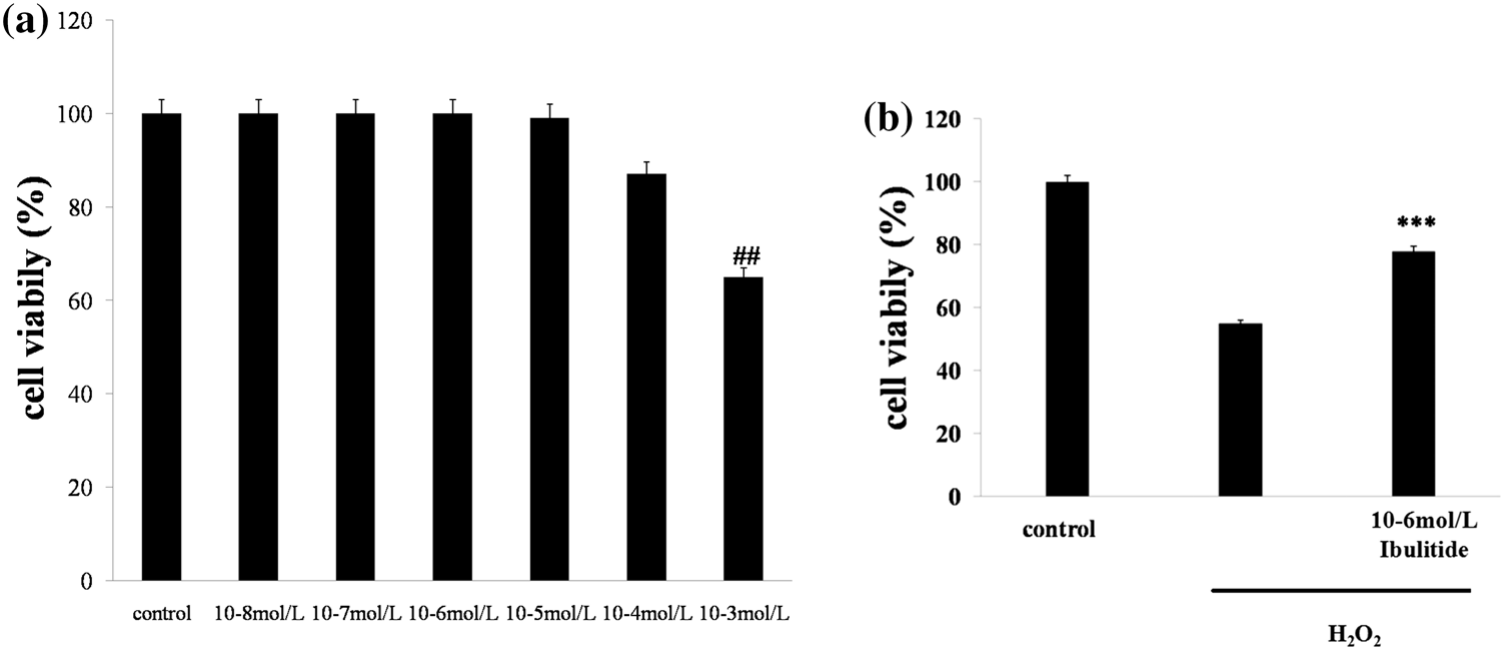
Effect of ibutilide on cell viability by MTT assay. **a**, **b** Effect of ibutilide on cardiomyocytes or H_2_O_2_-induced cardiomyocytes injury. All data are shown as mean ± SEM. ##*p* < 0.005 vs. Other ibulitide treatment group, ***p < 0.001 vs. H_2_O_2_ group, *n* = 6

After different concentrations (10^−8^ to 10^−3^ mol/L) of ibutilide-pretreated neonatal rat cardiomyocytes about 4 h, cell viability was detected using MTT. The results showed that the effect of ibutilide on neonatal rat cardiomyocytes emerged bi-directional with the concentration increase of ibutilide. Final concentration below 10^−8^ to 10^−3^ mol/L ibutilide caused little effects on cell proliferation. While the dose of ibutilide up 10^−4^ mol/L enhanced cytotoxicity (Fig. 1a). Then effects of ibutilide (10^−6^ mol/L) on H_2_O_2_-induced neonatal rat cardiomyocytes injury were analyzed by MTT. As shown in Fig. 1b, compared with control, the cell proliferation was markedly inhibited after H_2_O_2_ treatment, which was significantly improved by ibutilide.

### Ibutilide reduces the levels of MDA and increases the levels of SOD and GSH-px

**Fig. 2:**
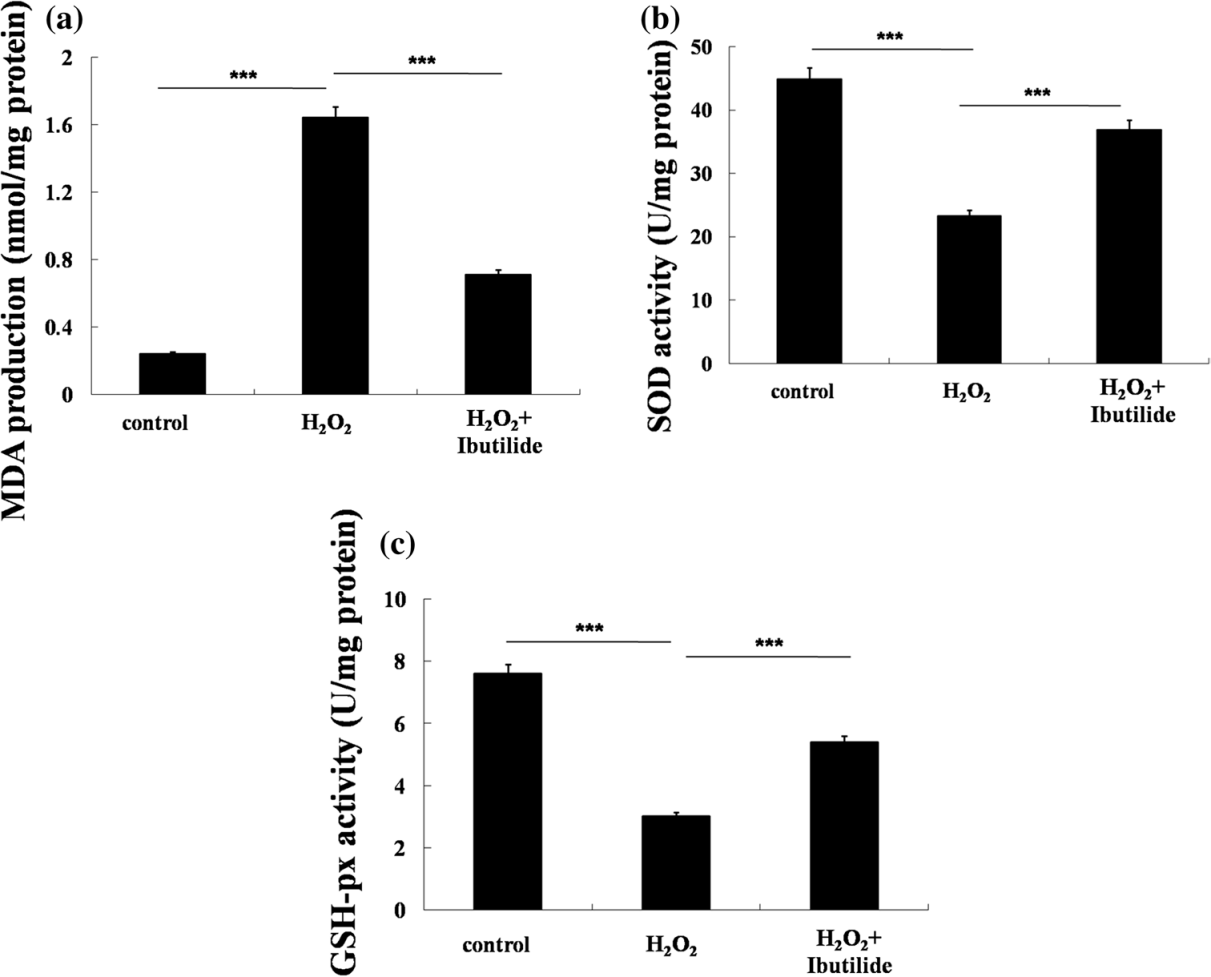
Ibutilide prevents H_2_O_2_-induced oxidative stress. **a**–**c** ELISA examines the level of MDA, SOD, and GSH-px. All data are shown as mean ± SEM. ***p < 0.001, *n* = 3

Physiological redox signaling is the role of reactive oxygen species (ROS) in intra- and intercellular communication [14]. When there is significant uncontrolled generation of ROS that overwhelm endogenous anti-oxidant capabilities, oxidative stress occurs; AF is associated with oxidative stress [15]. Cardiomyocytes were evaluated by ELISA. As an indicator of lipid peroxidation, the levels of MDA and SOD were analyzed (Fig. 2a, b). The levels of MDA were found to be higher in model group compared to control group, treatment with ibutilide significantly decreased the MDA levels. The levels of SOD were increased after treatment with ibutilide. The levels of GSH-px showed a significant reduction in model group compared with control group, while ibutilide increased the levels of GSH-px (Fig. 2c).

### Significant decreases apoptosis in the H_2_O_2_-treated cardiomyocytes after using ibutilide

**Fig. 3:**
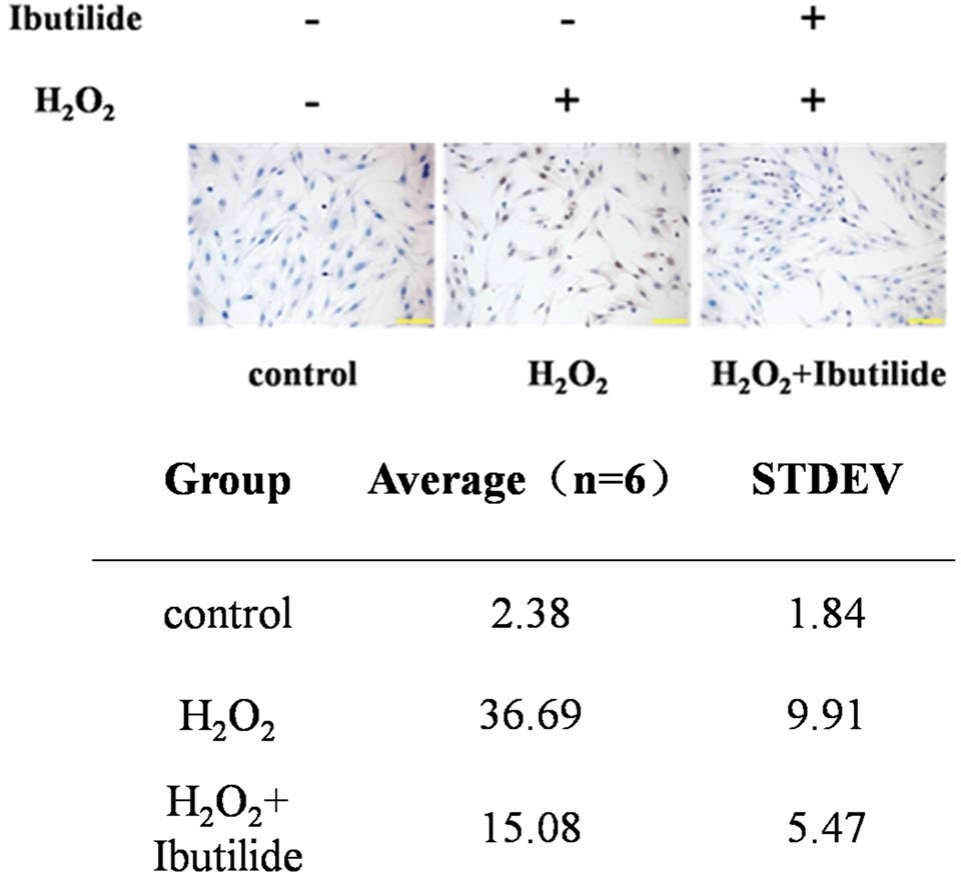
Apoptosis of cardiomyocytes was detected by TUNEL

Apoptosis plays an important role in AF [16]. To evaluate the effect of ibutilide on cardiomyocytes, the cells were treated by H_2_O_2_ with or without ibutilide and measurement of cell apoptosis using TUNEL analysis. Significant amounts of TUNEL-positive cells were observed in the H_2_O_2_-treated cardiomyocytes compared to the control cardiomyocytes. While the number of TUNEL-positive cells were decreased after dealing with ibutilide (Fig. 3).

**Fig. 4:**
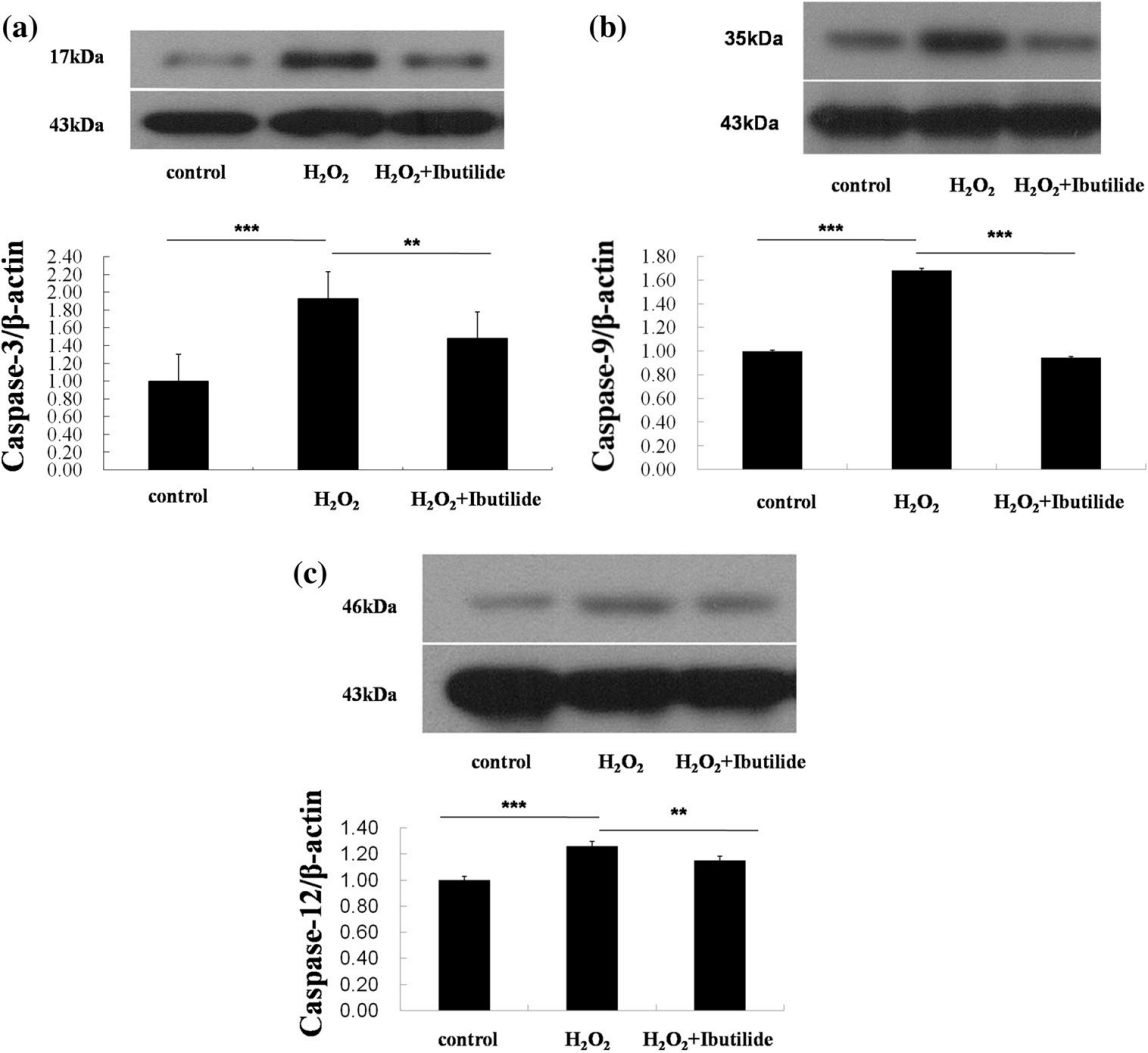
The expressions of Caspases were decreased by ibutilide. **a**–**c** Western blot analyze the expression of Caspase-3/9/12. All data are shown as mean ± SEM. **p < 0.05, *n* = 3; ***p < 0.001, *n* = 3

Apoptosis along with the activation of caspases was found in the myocardium of cardiomyopathy [17]. To confirm whether caspases were involved in the H_2_O_2_-induced apoptosis and the effect of ibutilide on the apoptosis, we tested the expression of Caspase-3, Caspase-9 and Caspase-12. The higher caspase activity was detected in the H_2_O_2_-treated cardiomyocytes when compared to the controls, while ibutilide inhibited caspase activity (Fig. 4).

### Ibutilide attenuated the apoptosis through mitochondria-dependent pathway in neonatal rat cardiomyocytes

**Fig. 5:**
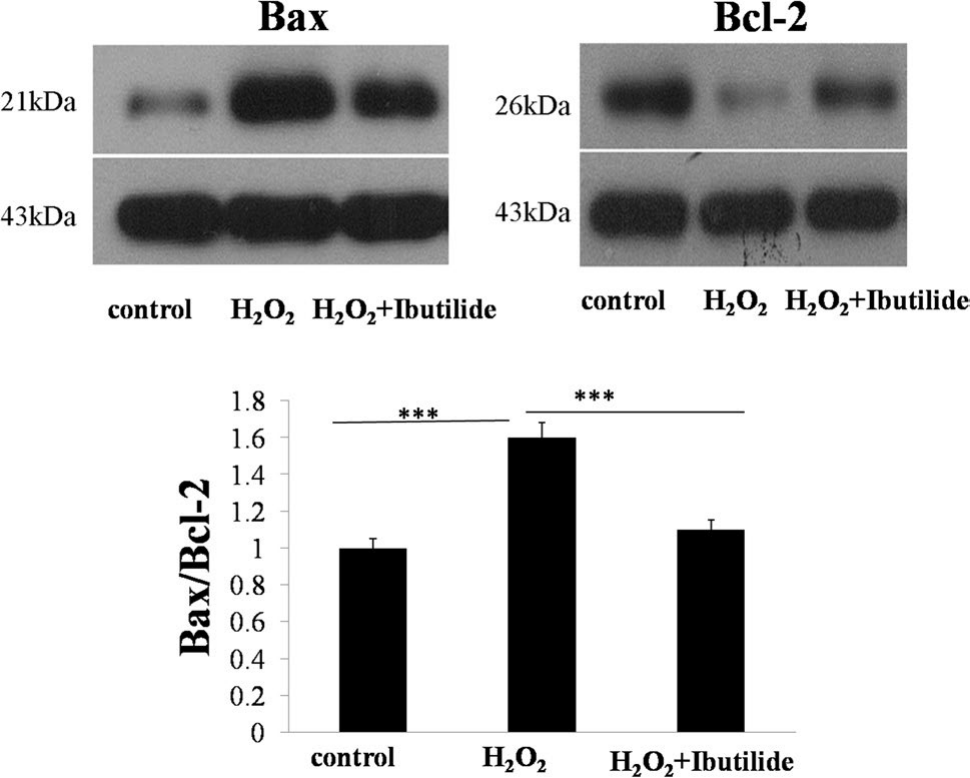
Western blot analyzed the expression of Bax, Bcl-2. All data are shown as mean ± SEM. ***p < 0.001, *n* = 3

Mitochondria-dependent pathway of apoptosis is regulated by Bcl-2 family members, such as pro-apoptotic protein Bax and anti-apoptotic protein Bcl-2 which are important for the caspase activation [18]. To confirm whether mitochondria-dependent pathway related with H_2_O_2_ induced apoptosis, we tested the expression of Bax and Bcl-2. In our study, the expression level of pro-apoptotic protein Bax (Fig. 5) in model group was significantly higher than in the control and treatment group, while anti-apoptotic protein Bcl-2 (Fig. 5) in model group did not.

### Ibutilide attenuated the apoptosis through ER-dependent pathway in neonatal rat cardiomyocytes

**Fig. 6:**
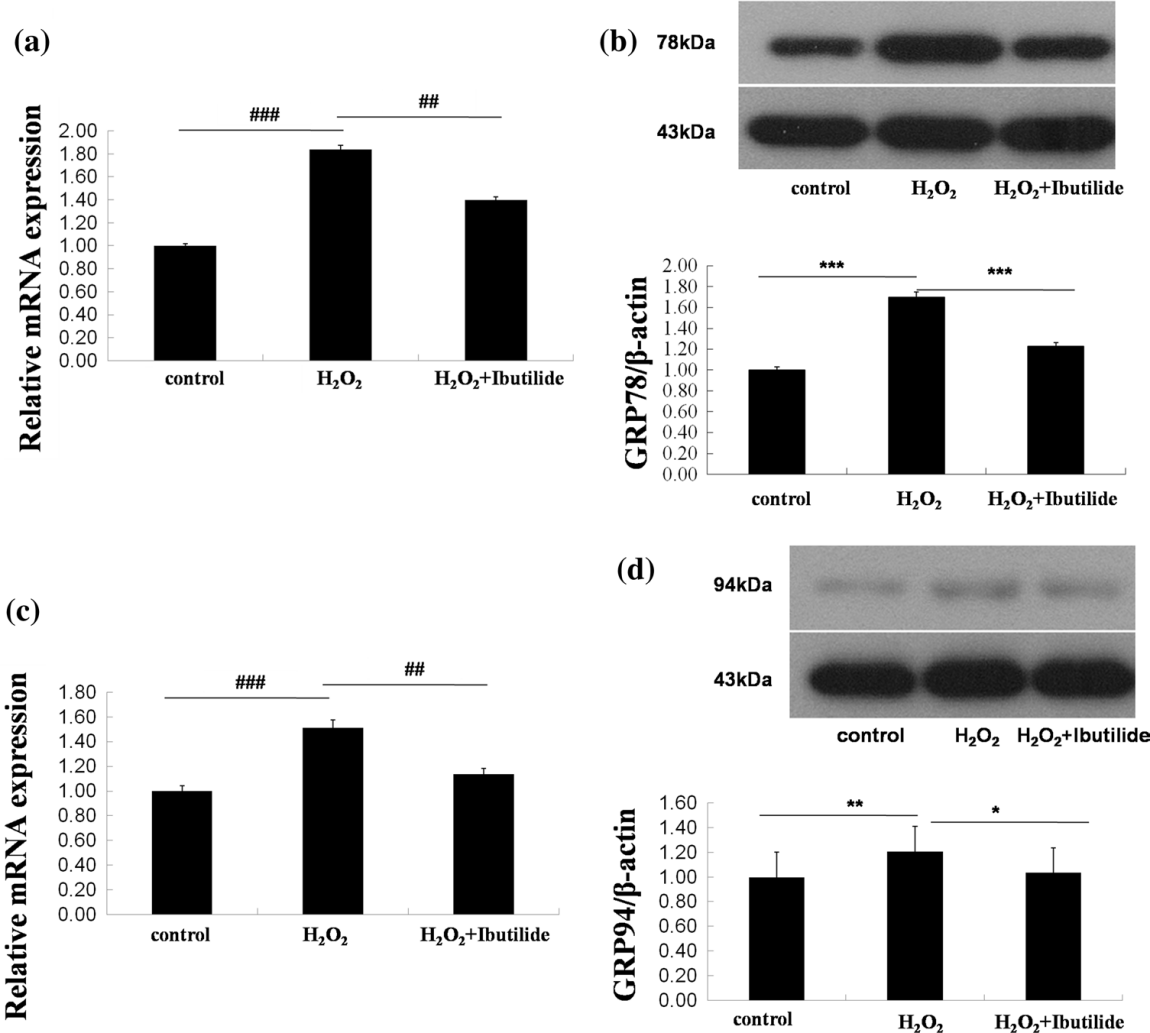
Ibutilide increased the expression of GRP78 and GRP94. **a**, **c** The mRNA were examined by qPCR. **b**, **d** The protein expressions were analyzed by WB. All data are shown as mean ± SEM. *p < 0.1 or #*p* < 0.1, *n* = 3; **p < 0.05 or ##*p* < 0.05, *n* = 3; ***p < 0.001 or ###*p* < 0.001, *n* = 3

ER stress-mediated apoptosis was also analyzed. ER is initiated by the GRP78 and GRP94 [19]. ER stress-associated genes (grp78, grp94) were analyzed by real-time PCR, and the level expressions of these proteins were evaluated by western blot. We have demonstrated grp78 or grp94 in model group were higher than control group and treatment group (Fig. 6a, c). The results of western blot were same with real-time PCR (Fig. 6b, d).

**Fig. 7:**
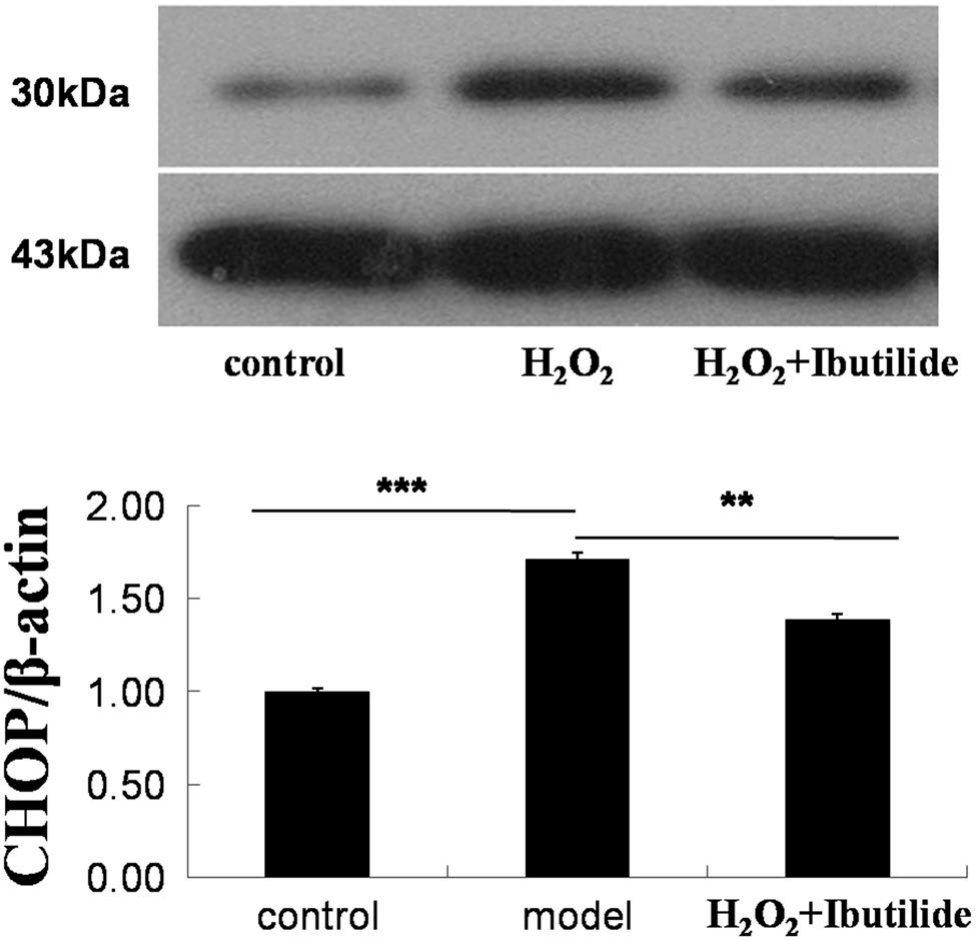
The expression of CHOP decreased by ibutilide. All data are shown as mean ± SEM. *p < 0.1, *n* = 3; **p < 0.05, *n* = 3; ***p < 0.001

CHOP related with ER stress [20]. We tested the expression of CHOP. Ibutilide reduced ER stress as indicated decreased level of CHOP (Fig. 7).

## Discussion

Atrial fibrillation is responsible for a significant health care burden due to its high and growing prevalence, incidence and potentially life-threatening complications [21]. The ability of ibutilide-treated atrial fibrillation has been widely acknowledged and is summarized [22]. In our study, we provide an evidence of potential therapeutic value of ibutilide-inhibited oxidative stress and apoptosis in neonatal rat cardiomyocytes subjected to H_2_O_2_ injury. The mechanisms of protection effects may be related with mitochondrial stress pathway and ER stress pathway.

Oxidative stress plays an important role in atrial fibrillation [23]. In cardiomyocytes, increased ROS has been found to be related with AF [24]. The increased level of ROS results in damage to proteins, lipids and DNA by augmenting cytokine production from activated inflammatory cells. ROS is involved in cardiac structural and electrical remodeling, all of which increase susceptibility to AF [23]. Our experiments determined the relative contribution of ibutilide to H_2_O_2_-stimulated cardiomyocytes. From the results, ibutilide resulted in a significant increase in MDA or SOD and decrease in GSH.

Apoptosis plays an important role in the pathogenesis of AF [25]. Some researchers have studied that cardiomyocyte apoptosis is related to oxidative stress [26]. In the present study, cardiomyocyte apoptosis was detected by TUNEL. Our results suggested that ibulitide could significantly reduce H_2_O_2_-induced apoptosis. Ibulitide also decreased the expression levels of rate of caspases.

The signal pathways through ibutilide exerted their effects that were analyzed. Mitochondrial stress pathway was researched. The mitochondria-dependent pathway for apoptosis is governed by Bcl-2 family proteins, such as Bax and Bcl-2 [27]. In our study, the ratio of Bax/Bcl-2 was analyzed by western blot. The increase in ratio of Bax/Bcl-2 in H_2_O_2_-induced neonatal rat cardiomyocytes indicated that the cells had undergone apoptosis by a mitochondria-dependent pathway, while these parameters decreased in the group treated with ibutilide. These results suggested that the ability of ibutilide to attenuated oxidative stress is partly by inhibiting the mitochondrial-related apoptosis.

Endoplasmic reticulum (ER) has been linked to many diseases, including AF. The accumulation of misfolded protein disrupts ER and leads to the activation of the classic coping mechanism termed the unfolded protein response [28]. This response is initiated by the GRP78 or GRP94. To investigate whether ibutilide would relieve ER damage, we examined grp78 and grp94 gene by real-time PCR and these proteins expression by western blot. From these results, we demonstrated a dose-related decrease in the mRNA expression of grp78 and grp94 after treated with ibutilide. These results suggested that ER stress could be relieved in H_2_O_2_-induced cardiomyocytes after treating with ibutilide. ER stress occurs for recover ER homeostasis of cell survival at a short time, while prolonged ER stress would activate apoptosis [29]. CHOP related to ER stress induces apoptosis [20]. Our results showed that the expression of CHOP was decreased after treated ibutilide.

**Fig. 8:**
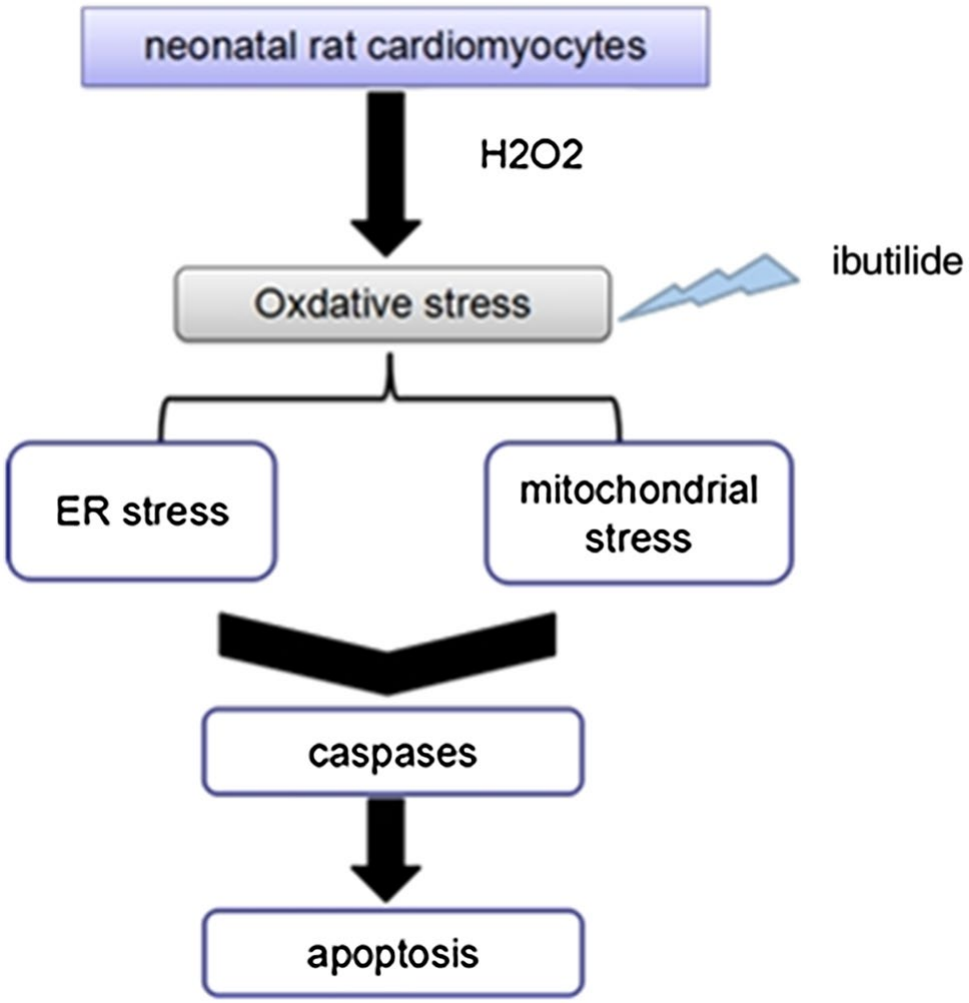
Schematic diagram of the signal pathways related with H_2_O_2_ induced apoptosis in neonatal rat cardiomyocytes

In conclusions, we provided in vitro evidence that ibutilide could protect H_2_O_2_-inducted oxidative stress and apoptosis, the mechanisms related with mitochondria-dependent pathways and ER stress-dependent pathways (Fig. 8).
